# Spatial Transcriptomics in Human Cardiac Tissue

**DOI:** 10.3390/ijms26030995

**Published:** 2025-01-24

**Authors:** Quynh Nguyen, Lin Wei Tung, Bruce Lin, Raam Sivakumar, Funda Sar, Gurpreet Singhera, Ying Wang, Jeremy Parker, Stephane Le Bihan, Amrit Singh, Fabio M.V. Rossi, Colin Collins, Jamil Bashir, Zachary Laksman

**Affiliations:** 1 Division of Cardiac Surgery, Department of Surgery, University of British Columbia, Vancouver, BC V6T 1Z4, Canada; 2 School of Biomedical Engineering, University of British Columbia, Vancouver, BC V6T 2B9, Canada; 3 Centre for Heart Lung Innovation, University of British Columbia, Vancouver, BC V6Z 1Y6, Canadaying.wang@hli.ubc.ca (Y.W.);; 4 Department of Medical Genetics, Pharmacology and Therapeutics, University of British Columbia, Vancouver, BC V6T 1Z4, Canada; 5 Department of Anesthesiology, Pharmacology and Therapeutics, University of British Columbia, Vancouver, BC V6T 1Z4, Canada; 6 Vancouver Prostate Centre, Vancouver, BC V6H 3Z6, Canada; 7 Department of Medicine, University of British Columbia, Vancouver, BC V6T 1Z4, Canada; 8 Department of Pathology and Laboratory Medicine, University of British Columbia, Vancouver, BC V6T 1Z4, Canada; 9 Division of Cardiology, Department of Medicine, University of British Columbia, Vancouver, BC V6T 1Z4, Canada; 10 Department of Urologic Sciences, University of British Columbia, Vancouver, BC V6T 1Z4, Canada; 11 St. Paul’s Hospital, Vancouver, BC V6Z 1Y6, Canada

**Keywords:** spatial transcriptomics, cardiovascular diseases, Visium, STomics, CurioSeeker, Xenium, GeoMX, CosMX, Molecular Cartography, MERSCOPE

## Abstract

Spatial transcriptomics has transformed our understanding of gene expression by preserving the spatial context within tissues. This review focuses on the application of spatial transcriptomics in human cardiac tissues, exploring current technologies with a focus on commercially available platforms. We also highlight key studies utilizing spatial transcriptomics to investigate cardiac development, electro-anatomy, immunology, and ischemic heart disease. These studies demonstrate how spatial transcriptomics can be used in conjunction with other omics technologies to provide a more comprehensive picture of human health and disease. Despite its transformative potential, spatial transcriptomics comes with several challenges that limit its widespread adoption and broader application. By addressing these limitations and fostering interdisciplinary collaboration, spatial transcriptomics has the potential to become an essential tool in cardiovascular research. We hope this review serves as a practical guide for researchers interested in adopting spatial transcriptomics, particularly those with limited prior experience, by providing insights into current technologies, applications, and considerations for successful implementation.

## 1. Introduction

The human heart is a highly complex organ composed of a heterogeneous population of cells that function in coordination. When this well-orchestrated system is disturbed, people develop cardiovascular diseases (CVDs). Despite advancements in sciences and medicine, CVDs remain the leading cause of death globally, contributing to 32% of all global deaths in 2019 [1]. Understanding cardiac cell biology, cell-cell interactions, and how cells interact with their microenvironment is essential to understand normal cardiac function and how these functions are altered in disease states. 

Traditional studies of cardiac physiology and pathology have been conducted using animal models, particularly rodents [2]. While studies using animal models have provided invaluable insights into the cardiovascular system, intrinsic differences in anatomy and physiology between animal models and humans prevent them from fully replicating the intricacies of human cardiac function. Other platforms, such as stem cell-derived models and cardiac organoids, are recent advancements contributing to our understanding of human heart biology [3,4]. However, they are limited by their ability to mimic the interconnected nature of cardiac tissue, where multiple cell types interact within a complex three-dimensional microenvironment. As a result, human tissue, although hard to come by, remains the best resource for studying human cardiac disease.

While traditional bulk omics approaches have provided invaluable understanding of the underlying cardiac pathophysiology, they only measure the average gene expression and fail to detect the heterogeneity within cardiac tissue. Single-cell RNA sequencing (scRNA-seq) and single-nuclei RNA sequencing have revolutionized the field by providing information on gene expression and regulation at the single-cell level. For simplicity, both methodologies will be collectively referred to as single-cell RNA sequencing (scRNA-seq) throughout this paper. ScRNA-seq technologies have revealed a comprehensive atlas of the human heart by identifying rare cell populations and their gene signatures, which are instrumental in advancing our understanding of cardiac development and disease [5].

Despite significant advancements in scRNA-seq, one critical limitation with this technology remains: the lack of spatial context. ScRNA-seq requires the dissociation of tissue, which disrupts the connections between cells and their extracellular environment. However, no cell exists in isolation, and the function and regulation of each cell are determined by its interaction with neighboring cells and its microenvironment. Spatial transcriptomics (ST) bridges this gap by providing high-resolution data while preserving the spatial context of tissue. This novel approach offers invaluable insights into the molecular signatures underlying cardiac tissue in both healthy and diseased states.

This paper aims to review the current technologies in spatial transcriptomics with a focus on commercially available platforms, discuss their applications in cardiac research using human tissue, evaluate associated challenges and limitations, and provide insights into emerging opportunities in the field. 

## 2. Spatial Transcriptomics: Current Technologies, Platforms, and Experimental Considerations

**Figure 1:**
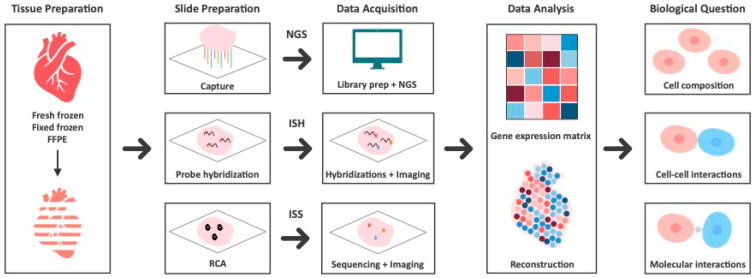
Workflow of spatial transcriptomics, outlining tissue preparation, slide preparation, data acquisition, data analysis, and downstream biological applications. The process begins with tissue preparation from fresh frozen, fixed frozen, or formalin-fixed paraffin-embedded (FFPE) samples. Sequencing-based methods use spatial barcodes to preserve spatial locations of transcripts and next-generation sequencing (NGS) to identify transcripts. In situ sequencing (ISS) relies on rolling circle amplification (RCA) to anchor and amplify complementary DNA (cDNA) while retaining the spatial context. Fluorescently labeled oligonucleotide probes then hybridize to cDNA using sequencing by ligation. In situ hybridization (ISH) methods detect target sequences by hybridization of complementary fluorescent probes. The product of spatial transcriptomics is the gene expression matrix with spatial reconstruction to address key biological questions such as cell composition, cell-cell interactions, and molecular interactions. FFPE, formalin-fixed paraffin-embedded; ISH, in situ hybridization; ISS, in situ sequencing; NGS, next-generation sequencing; RCA, rolling circle amplification.

Spatial transcriptomics seeks to quantify the number of gene transcripts at specific spatial positions within a tissue. Multiple methods exist, which differ in terms of capture area size, resolution, and number of target genes [6,7,8]. Table 1 highlights key parameters of currently available commercial ST platforms. There is often a trade-off between the number of genes that can be analyzed and the size of the tissue that can be captured. Depending on how the spatial location is retained and how RNA is quantified within each location, ST technology can be broadly categorized into two main methods: sequencing-based and imaging-based technologies [9,10]. Sequencing-based approaches use spatial barcodes to map the spatial position of each transcript and next-generation sequencing followed by alignment to a reference genome, generating a gene expression matrix [10]. Imaging-based approaches image RNA in situ via microscopy. Within imaging-based technologies, there are two ways to distinguish different RNA species: hybridization, where RNA species are hybridized to fluorescent probes (in situ hybridization or ISH), or in situ sequencing (ISS), where transcripts are directly sequenced inside a tissue section using sequencing by ligation (SBL) technology [11,12,13]. Figure 1 illustrates the workflow of these ST technologies from tissue preparation to the generation of spatially-resolved gene expression matrix. Clearly, this classification is not always distinct, as methods may combine elements from multiple technologies.

### 2.1. Imaging-Based Technologies

ISH-based methods build upon the foundation of single-molecule fluorescence in situ hybridization (smFISH), a technique where nucleic acid sequences are labeled with complementary fluorescent probes, allowing individual transcripts to appear as distinct spots under a microscope [14]. Expanding on smFISH, ISH-based technologies use multiple rounds of hybridization in order to profile a larger number of genes. Sequential fluorescence in situ hybridization (seqFISH) is a technique that utilizes iterative rounds of hybridization, each time with a different fluorophore [15]. Between each round, fluorescent signals are captured, stripped, and replaced with a new set of fluorophores, generating unique fluorescent signatures specific to each gene. Molecular Cartography (MC), a spatial transcriptomics platform developed by Resolve Biosciences, leverages the principles of seqFISH to detect transcripts with high sensitivity and specificity. However, seqFISH is practically limited by optical crowding if too many transcripts are profiled simultaneously [16]. Currently, the MC platform can detect up to 100 genes with subcellular resolution, suitable for analysis of rare transcripts in the cells.

Each round of hybridization in ISH-based methods carries a risk of error due to factors such as non-specific bindings, mismatches, and probe degradation. The likelihood of errors increases exponentially as more rounds are performed. To address this problem, multiplexed error-robust fluorescence in situ hybridization (MERFISH) employs a different strategy to reduce such errors [17]. MERFISH also relies on sequential hybridization; however, the technology uses binary-coded secondary probes that are either fluorophore-labelled or unlabeled [18,19,20]. Successive rounds of hybridization are imaged to detect the presence or absence of fluorescent signals, and transcript identities are decoded using error-robust barcodes, which help detect and correct errors during hybridization [17]. Furthermore, this approach also mitigates optical crowding, allowing for a higher number of transcripts to be processed at the same time [20]. MERFISH-based technology is commercially available through platforms like MERSCOPE from Vizgen, which can image up to 1000 genes with subcellular resolution and capture an area of up to 3 cm². Likewise, the CosMx Spatial Molecular Imager (CosMx SMI) from Nanostring uses a hybridization strategy similar to MERSCOPE in principle. Current CosMx SMI supports the imaging and quantification of up to 6000 genes at subcellular resolution.

ISS-based methods enable the direct readout of transcript sequences within tissue samples, preserving their spatial information. The process begins with RNA being reverse-transcribed into cDNA, followed by rolling circle amplification (RCA), which anchors and amplifies the cDNA while retaining its spatial context [21]. Subsequently, fluorescently labeled oligonucleotide probes hybridize to the RCA-amplified cDNA at complementary sequences using DNA ligase, a process called sequencing by ligation. This step enables visualization and eventually identification of RNA species at their spatial locations. An application of ISS is CARTANA technology, which was acquired by 10x Genomics and further developed into the current Xenium platform. 

The Xenium platform integrates both ISS and ISH technologies to retain spatial context and identify RNA species. The workflow begins with gene-specific barcoded probes hybridized directly onto RNA in the tissue using the proprietary Xenium slide. Xenium’s padlock probes provide highly specific target detection by requiring both probe arms to hybridize to the target sequence. If only one arm hybridizes, the probe remains unstable and will be washed off in the post-hybridization wash. Targeted RNAs are then amplified via RCA followed by sequential rounds of hybridization using fluorophore-labeled oligonucleotide probes. This iterative imaging process generates a unique imaging signature, allowing accurate gene identification. The Xenium platform supports the detection of up to 5000 genes with subcellular resolution, covering a capture area of 22.5 mm × 10.5 mm, making it a powerful tool for spatial transcriptomic studies. 

### 2.2. Sequencing-Based Technologies

Sequencing-based spatial transcriptomics techniques extend the principles of scRNA-seq by introducing spatial barcoding prior to library preparation [10]. These methods use spatially barcoded slides to capture polyadenylated RNA, preserving the spatial location of transcripts during reverse transcription [22,23]. The RNA species are then identified through next-generation sequencing (NGS), allowing whole transcriptome profiling. An example of this technology is Visium by 10x Genomics, which enables whole transcriptome analysis with near single-cell resolution over a capture area of 6.5 mm × 6.5 mm.

An alternative to spatial barcodes involves depositing barcoded beads onto a slide for mRNA capture, as demonstrated in the Slide-seq method [24]. Spatial information of each random barcode is determined through in situ indexing. Platforms like CurioSeeker by Curio Bioscience employs bead-based technology, supporting whole-transcriptome analysis over capture areas of 3 mm × 3 mm or 10 mm × 10 mm. Another advanced sequencing-based approach is Stereo-seq (spatial enhanced resolution omics-sequencing), which uses randomly barcoded DNA nanoballs arranged in an array to achieve nanoscale resolution [25]. The STomics platform, utilizing Stereo-seq technology, facilitates whole-transcriptome profiling with subcellular resolution in fresh frozen tissues, accommodating capture regions as large as 13 cm × 13 cm. These innovations provide versatile and scalable solutions for spatial transcriptomics, catering to a range of experimental needs and resolutions.

The GeoMx Digital Spatial Profiler (DSP), an ST platform developed by Nanostring Technologies, uses a hybrid strategy combining aspects of sequencing-based and imaging-based ST approaches. The typical workflow using this platform includes hybridization of gene-specific probes to RNA targets on the tissue sample. These probes are linked to unique barcodes through UV-cleavable linkers. The tissue slide is then stained with fluorescently labeled imaging probes to visualize specific cell types of interest, followed by imaging with the GeoMx DSP instrument. Based on the imaging results, researchers can select regions of interest (ROIs) for further analysis. UV light is then applied to these ROIs to release the barcodes, which are subsequently collected for library construction and sequencing. This targeted library preparation is more cost-effective. Furthermore, more samples on the same slide can be processed without being limited by predesigned capturing areas as in other array-based platforms [26]. 

### 2.3. Considerations for Selecting a Spatial Transcriptomic Method

#### 2.3.1. Biological Question: Hypothesis Testing vs. Hypothesis Generation

The choice of ST method depends largely on whether the study is hypothesis generating or hypothesis testing. Sequencing-based approaches are well-suited for hypothesis generation because they capture all polyadenylated transcripts, providing an unbiased view of the entire transcriptome. Imaging-based methods, on the other hand, are often preferred for hypothesis testing since they typically require prior knowledge of target genes. However, advancements in imaging-based technologies now allow profiling of up to 6000 genes, thus broadening their applications.

An important consideration is the trade-off between sensitivity and gene throughput. Imaging-based methods generally offer higher sensitivity compared to sequencing-based methods, which achieve their unbiased whole transcriptomic coverage at the expense of sensitivity. For imaging-based platforms, the number of genes to be profiled is another factor to consider. Technologies like MC by Resolve Biosciences can profile up to 100 genes, while platforms such as Xenium by 10x Genomics can profile up to 5000 genes.

#### 2.3.2. Species and Tissue Compatibility

The species and tissue types available for the study also play an important role in the process of platform selection. Most commercially available ST platforms are compatible with human and mouse tissues. Researchers also need to consider the specific tissue types available for their experiment—whether fresh frozen, fixed frozen, or formalin-fixed paraffin-embedded (FFPE)—as each platform is compatible with certain tissue types. For FFPE tissues, RNA quality is an additional consideration. Some platforms recommend assessing RNA integrity prior to proceeding with the workflow, often suggesting that more than 50% of RNA fragments be above 200 nucleotides for optimal results.

#### 2.3.3. Tissue Size

The size of the tissue to be analyzed is another important factor, as all platforms have constraints based on their capture area sizes. For example, 10x Visium supports tissue sections of up to 6.5 mm × 6.5 mm, with two sections per slide. In comparison, 10x Xenium offers a larger capture area of 22.5 mm × 10.5 mm per slide, with two slides processed per run. Meanwhile, STomics’s Stereo-seq accommodates significantly larger tissue sections, up to 13 cm × 13 cm, making it ideal for studies requiring larger spatial coverage.

#### 2.3.4. Spatial Resolution

Imaging-based methods generally offer better resolution than sequencing-based methods, often achieving subcellular resolution. Among sequencing-based platforms, 10x Visium is the most commonly used platform. Visium’s resolution has improved to almost single-cell scale, with data output at 2 × 2 µm, although the recommended starting bin size for analysis is 8 × 8 µm. Bins provide the benefit of containing a higher average number of transcripts per unit area. An 8 µm bin captures 16 times more transcripts compared to a 2 µm square, therefore enhancing the signal-to-noise ratio while preserving single-cell scale resolution. Sequencing-based approaches are often combined with scRNA-seq to achieve single-cell resolution while preserving all the advantages that NGS technology can offer.

Overall, designing an ST experiment involves balancing sensitivity, resolution, and tissue compatibility with the study’s biological question, tissue type, and size availability. Beyond these considerations, adhering to best practices is important to ensure reliable results. First of all, RNA integrity is essential for a successful ST experiment. Tissue handling and preparation should follow platform-specific guidelines, including proper fixation, freezing, and sectioning with RNA quality checks before processing. Maintaining an RNase-free lab space dedicated to ST work is critical [27]. Furthermore, the retrieval, collection, and storage of tissue samples significantly influence RNA quality. Proper training and communication are essential to limit ischemic time and reduce contamination risks during sample handling.

Integrating spatial transcriptomic data with histological information such as hematoxylin and eosin staining can provide additional spatial context, allowing for the correlation of molecular data with tissue architecture. Preservation of spatial orientation across tissue samples is equally crucial to ensure consistency among experimental groups. Researchers should always consult vendor protocols and platform-specific guidelines to align workflows with established standards in order to optimize their results. 

## 3. Spatial Transcriptomics Data Analysis

The analysis of spatial transcriptomic data can be divided into two phases: pre-processing and downstream data analysis. Multiple previous papers have comprehensively reviewed the data analysis workflows for ST [6,7,26,28]. In this paper, we aim to provide an overview of the key steps and tools available for each step.

### 3.1. Pre-Processing

Pre-processing is the first step in ST analysis and involves converting raw imaging or sequencing data into a matrix of transcript counts by spatial capture areas. For image-based data, this step identifies location, type, and expression levels of RNA within the tissue image. Corrections for variations in background and noise are needed to ensure data accuracy. Various software packages are used for this purpose, such as DeepBlink, BarDensr, or graph-ISS [29,30,31]. 

For sequencing-based data, pre-processing begins with tissue image registration, where the tissue area is divided into spatial spots to generate a location index matrix corresponding to each region. A gene expression matrix is created by aligning sequenced reads to a reference genome. These two matrices are then combined to produce a spatially indexed transcript count matrix. A commonly used tool for this workflow is SpaceRanger from the 10X Visium platform.

Segmentation is an optional step in spatial transcriptomic data analysis. Segmentation aims to reconstruct single-cell transcriptomes from ST data with subcellular resolution. This approach utilizes references from scRNA-seq data, previous information from nuclear staining, or inference based on clustering of transcript species. Tools like Baysor, Sparcle, or Spage2vec are commonly used for this purpose [32,33,34]. Segmentation ensures that ST data retains the structural context needed for meaningful downstream analysis.

Normalization is an essential step in pre-processing and addresses technical variability across spatial spots due to differential mRNA capture rates across the tissue. The most common normalization approach divides each spot’s gene expression values by the total transcript count in the spot, as implemented in tools such as Scanpy, Giotto, or Seurat [35,36,37]. However, this approach uses the assumption that all regions within the tissue have uniform mRNA abundance, which may not always be true. Other methods, such as the sctransform algorithm in Seurat or the spatial and morphological expression (SME) method in stLearn, account for spatial context and variability in tissue composition [38]. Interestingly, unnormalized data can also provide valuable insights, as information on cell density and mRNA abundance is preserved; however, their suitability for downstream analysis may be limited [38].

### 3.2. Downstream Data Analysis

#### 3.2.1. Dimensionality Reduction and Clustering

Dimensionality reduction and clustering are foundational methods for identifying distinct spatial regions within tissues. Dimensionality reduction techniques such as principal component analysis (PCA) or uniform manifold approximation and projection (UMAP) are utilized to reduce data noise and highlight key biological patterns, facilitating downstream clustering [39,40]. Spatial clustering uses spatial transcriptomic information to categorize tissue locations into multiple domains. Machine learning-based clustering algorithms such as Louvain clustering, Leiden clustering, or k-means clustering are commonly used for this purpose [28].

#### 3.2.2. Integration with Single Cell RNA Sequencing Data

Spatial cell type annotation incorporates scRNA-seq data with ST data to identify and label specific cell types or states within the tissue. This integration bridges the high-resolution cellular data from scRNA-seq with spatially resolved expression patterns from ST data. Methods such as imputation, deconvolution, and mapping achieve this integration. Imputation involves predicting missing or unobserved values in ST datasets by using information from neighboring spots or scRNA-seq references. This step essentially “fills in the gaps”, thereby enhancing spatial resolution, particularly for methods with low sensitivity. Deconvolution, on the other hand, uses machine learning, deep learning, and statistical models to estimate the proportion of different cell types or cell states within spatial spots. This step is important in methods that do not reach single-cell resolution, as each spatial spot may contain multiple cells. Mapping assigns scRNA-seq-derived cell types or states to individual spatial spots by aligning known cellular annotations from scRNA-seq data to ST profiles. This mapping step is commonly used for data generated via image-based technologies. Examples of platforms for these steps include Seurat or Giotto [36,37].

#### 3.2.3. Interaction Analysis

A common goal of transcriptomic studies is to infer cellular and molecular interactions. Similar to scRNA-seq data analysis, there are multiple software packages that are designed for this purpose. Cell-cell interaction analysis examines how cells interact spatially, integrating spatial metrics with gene expression data. Tools such as SpOTsc and DIALOGUE facilitate this type of analysis [41,42]. Gene-gene interaction analysis explores molecular mechanisms underlying cellular interactions by using gene expression and positional matrices. Methods like GCNG and ScHOT are used to analyze gene co-expression patterns and their spatial relationships [41,42]. Although numerous packages are available, they primarily differ in functionality and the size of their user communities. For novice researchers, Seurat and Scanpy are highly recommended due to their comprehensive documentation and active, supportive user bases.

## 4. Applications of Spatial Transcriptomics in Human Cardiac Research

The following section reviews key studies that have used ST on various human cardiac tissues. These papers demonstrate the diverse applications of ST in uncovering spatial gene expression patterns and their implications in disease mechanisms. Table 2 summarizes the studies, with a focus on the techniques, platforms, and associated methods used in each.

### 4.1. Cardiac Development

Asp et al. used a combination of three technologies: NGS-based ST, scRNA-seq, and ISS-based ST to study the spatial and temporal differential gene expression patterns during human heart development [43]. The authors were able to characterize different developmental stages at an organ-wide level with single-cell resolution. NGS-based ST was used to explore spatial genome-wide expression patterns common to the three stages: 4.5–5, 6.5, and 9 weeks post-conception (WPC). This analysis was conducted using the method described by Stahl et al. in 2016, which was later commercialized as the Visium platform by 10x Genomics [53]. 

To delineate cell type heterogeneity, scRNA-seq was then applied to the intermediate developmental stage using the 10x Genomics platform. Results from NGS-based ST and scRNA-seq revealed distinct cell populations and their unique expression profiles. Building on these results, as well as prior knowledge of critical genes for cardiac embryogenesis, the group designed a custom panel of 69 genes and used ISS-based ST to further map cellular expression with subcellular resolution. The protocol was based on the method described by Ke et al. in 2013 using RCA and ISS [21]. Validation of these findings was performed using smFISH. 

The study revealed that spatial gene expression patterns are established early on and maintained across three stages. Additionally, distinct spatiotemporal expression patterns were identified in various cell populations, such as cardiac neural crest cells and Schwann progenitor cells, and new cell types involved in heart development were discovered. Findings from this study were used to create a publicly accessible web resource for the human embryonic heart, providing a valuable tool for future research in the field. 

More recently, Farah et al. utilized scRNA-seq and MERFISH to further investigate the development of the human heart [44]. The study focused on human hearts between 9 and 16 WPC. For scRNA-seq, each heart was dissected into chambers and interventricular septum before the 10x Genomics platform was used, which revealed 12 major cell classes, 39 populations, and 75 subpopulations corresponding to their anatomical locations and developmental stages. MERFISH was then performed on hearts from 12–13 WPC to spatially resolve the findings from scRNA-seq. A list of 238 genes was selected based on scRNA-seq results and prior knowledge. Using FFPE samples, MERFISH results provided high-resolution spatial mapping of individual cells, enabling the generation of a detailed cardiac cell atlas.

Among the key findings, ventricular cardiomyocyte subpopulations were found to have an unexpectedly complex organization across the ventricular wall. Furthermore, distinct ligand-receptor signaling pairs expressed between spatially neighboring cell populations were identified. In particular, plexin-semaphorin (PLXN-SEMA) signaling pathways were found to be essential in directing multicellular interactions during ventricular wall morphogenesis. The role of PLXN-SEMA signaling was further explored using in vitro pluripotent stem cell models and mouse models. These experiments revealed previously uncharacterized multicellular interactions among PLXNA2^+^ PLXNA4^+^ ventricular cardiomyocytes, SEMA3C^+^ SEMA3D^+^ fibroblasts, and SEMA6A^+^ SEMA6B^+^ endothelial cells, which potentially control the allocation of cardiomyocytes during ventricular wall compaction.

Another study by Lazar and colleagues also utilized ST and scRNA-seq to study the spatial dynamics of the developing human heart [45]. Using NGS-based ST on sixteen fresh-frozen human hearts collected between the 6th and 12th WPC, thirty-eight heart sections were generated with at least two biological replicates per sample. These sections were analyzed using the Visium platform, revealing previously unappreciated transcriptomic signatures in the developing heart, especially in the papillary muscles and atrioventricular regions. 

To complement the ST data, ISS was performed on five hearts targeting 150 selected transcripts. The protocol used for ISS was based on the previously published pipeline by Lee et al. [54]. In addition, to deconvolute the ST data and gain better resolution, scRNA-seq was performed on 15 hearts using the 10x Genomics platform. This analysis identified 31 coarse and 72 fine-grained cell states, offering further insight into the cellular landscape of the developing human heart.

By integrating ST and scRNA-seq, Lazar et al. were able to find novel insights into the development of multiple cardiac components, such as pacemaker conduction system, cardiac autonomic innervation, and structural regions such as the heart valves or atrial septum. The study also delineates the heterogeneity of the cardiac fibroblast population and presents the first spatial account of chromaffin cells in the fetal human heart. 

### 4.2. Cardiac Electro-Anatomy and Immunology

A study conducted by Kanemaru et al. combined ST and single-cell multiomics to investigate the cellular architecture of the human heart [46]. Using flash-frozen, optimal cutting temperature (OCT)-frozen, and FFPE tissues, ST was performed across eight cardiac anatomical regions using the Visium platform. The data were integrated with scRNA-seq data and single-nucleus assay for transposase-accessible chromatin sequencing (snATAC-seq) to analyze cellular profiles as well as the regulatory mechanisms governing cardiac cell identity. Key findings were verified using human-induced pluripotent stem cell-derived cardiomyocytes (hiPSCs). 

The study provided insights into the human cardiac conduction system, identifying distinctive ion channels, G protein receptors, and regulatory networks specific to pacemaker cells with FOXP2 found to be important in their phenotype. The sinoatrial node was found to be compartmentalized, with a core of pacemaker cells surrounded by fibroblasts and glial cells that support glutamatergic interactions. Furthermore, the study also identified immune niches in the epicardium that are enriched in both IgA and IgG that may contribute to infection defense. Another highlight of the study was the development of drug2cell, a platform that integrates single-cell profiles with drug-target databases to predict drug effects on specific cardiac cell types.

A recent study published in 2024 by Vyas and colleagues investigated the role of tissue-resident memory T (T_RM_) cells in the epicardial adipose tissue (EAT) and their contribution to atrial fibrillation (AF) [47]. Initial results from flow cytometry identified an enrichment of T_RM_ cells in patients with AF. To confirm the identity of these T_RM_ cells, cellular indexing of transcriptomes and epitopes by sequencing (CITE-seq) was used to delineate both transcriptomes and surface protein expression, a function that cannot be achieved by scRNA-seq alone. CITE-seq revealed two distinct T_RM_ populations differentiated by activation states and effector functions. Single-cell T cell receptor sequencing then showed significant clonal expansion and overlap between EAT and atrial tissue, suggesting that T_RM_ cells migrate between the two regions.

Next, ST, using the GeoMx DSP platform on FFPE tissues, revealed the border zone between EAT and the atrium to have intense inflammation and fibrotic activity, with upregulation of multiple pro-inflammatory cytokines and genes associated with tissue remodeling. The findings were validated in vitro by co-culturing human iPSCs with T_RM_ cells, which demonstrated that T_RM_ cells alter calcium flux, as well as inflammatory and apoptotic signaling pathways. The study offers insights into the immune-driven mechanism underlying AF, uncovering potential pathways for therapeutic interventions. 

Researchers can leverage previously generated ST datasets to enhance their own studies, as demonstrated in the paper by Amrute et al. [48]. This study investigates the role of cardiac fibrosis in heart failure using a multiomics approach on human cardiac tissues from 45 healthy donors, acutely infarcted, and chronically failing human hearts. ScRNA-seq, ATAC-seq, and CITE-seq were employed for comprehensive cellular and epigenetic characterization. Incorporating ST data from a previous study, the authors found a fibroblast trajectory marked by fibroblast activator protein (FAP) and periostin (POSTN) expression, which was governed by inflammatory cytokines derived from monocytes and macrophages [49]. Mesenchyme homeobox 1 (MEOX1) was also identified as an important transcription factor in the pathogenesis of cardiac fibrosis.

An important aspect of the field is the selection of appropriate experimental models to study human cardiac fibroblasts and fibrosis. Using scRNA-seq, the study found that in vivo mouse models contain many of the fibroblast populations found in the human heart and are better than cultured human primary cardiac, dermal, or immortalized fibroblasts. Furthermore, cell interaction analysis identified enrichment of interleukin-1 beta (IL-1β) and transforming growth factor beta (TGF-β) signaling in fibroblasts in heart failure. IL-1β was found to be selectively expressed by C-C chemokine receptor 2 (CCR2^+^) monocytes and macrophages in the human heart. This was confirmed with an in vivo study, which showed that IL-1β signaling from CCR2^+^ macrophages to fibroblasts is causally linked to cardiac fibrosis. The study highlights the therapeutic potential of modulating immune–fibroblast communication in the treatment of heart failure. 

### 4.3. Ischemic Heart Disease

Kuppe et al. constructed a comprehensive spatial multiomics map of human myocardial infraction using scRNA-seq, scATAC-seq, and NGS-based ST [49]. By profiling human tissues from control and infarcted hearts, the authors identified unique cell types and states and signaling pathway activities across the spectrum of cardiac tissue zones. Integrating these findings with ST, the study found multiple regulatory networks that drive the tissue remodeling process post-myocardial infarction, identifying important transcription factors such as myocyte enhancer factor 2D (MEF2D) and nuclear receptor subfamily 3 group C member 2 (NR3C2) that regulate cardiomyocyte stress states and runt-related transcription factor 1 (RUNX1) in fibroblast activation. Pathways such as transforming growth factor beta (TGFβ) and nuclear factor kappa B (NFκB) were also found to play essential roles in fibrotic and inflammatory processes. 

The multiomic data revealed the transition from injured cardiomyocytes to stressed states, reflecting their proximity to inflammatory zones. In addition, fibroblast-to-myofibroblast differentiation was found to be important in fibrosis and late-stage remodeling after myocardial infarction. Furthermore, endothelial cell subtypes were resolved with specific spatial roles in vascular remodeling and immune modulation. The main findings of the study were validated through in vitro and in vivo studies, confirming the role of key pathways and regulatory factors in post-myocardial infarction remodeling. Overall, this study provides a comprehensive map to the pathogenesis of human myocardial infarction, paving the way for further mechanistic and therapeutic studies in the field. ST datasets generated from this study have been used in other research on myocardial infarction and heart failure, allowing other researchers to integrate these data into their studies without the need to repeat often costly experiments [48,51].

An example of how previous ST datasets can be incorporated into new studies is demonstrated in the work by Ninh et al. [51]. Reanalyzing human ST data together with generating their own multiomics data from mice, the authors discovered novel spatially clustered interferon-induced cell colonies (IFNICs) in the infarct border zones. Importantly, cardiomyocytes were found to be the main drivers of this process, which was proposed to be mediated by interferon regulatory factor 3 (IRF3). This work highlights how existing human ST datasets can provide new insights into disease mechanisms without the need to replicate resource-intensive experiments. 

A recent study by Linna-Kuosmanen and colleagues investigated human ex vivo right atrial (RA) tissue from 39 patients with ischemic heart disease at multiple stages of progression to heart failure, along with 10 controls [50]. Using scRNA-seq and sequencing-based ST through Visium, as well as imaging-based ST through Molecular Cartography, the study identified pro-inflammatory microvascular dysfunction and changes in RA tissue composition as critical factors in disease progression. 

Mapping of vascular cell subtypes in human ex vivo RA tissues revealed differences in gene and spatial expression across various cell types and disease states. Transcription factor Krüppel-like factor 2 (KLF2) was found to play an important role in maintaining homeostasis in vascular endothelial cells. Further analysis showed metabolic reprogramming in both ischemic heart failure and non-ischemic heart failure driven by IL-1β. Chronic inflammation was marked by the accumulation of immune cells, in particular, macrophages. Two populations of lipid-associated macrophages were identified, with each population closely linked to ischemic heart disease and non-ischemic heart failure. Analysis of paired tissue and pericardial fluids indicated the association of interferon-responsive macrophages with advanced disease and chronic inflammation state. 

The study also demonstrated how disease-associated genetic variants affect disease processes across cell types. Two key genes, supervillin (SVIL) and junctional cadherin 5 associated (JCAD), were identified as examples of how such variants can modulate gene expression in multiple cells. The integration of scRNA-seq and ST provided a detailed cellular and spatial map of RA tissue in various disease stages. Furthermore, this study also underscores the importance of extending research on human heart diseases beyond the most apparent sites of pathology to include functionally critical but less-studied regions.

Another study by Gastanadui et al. used GeoMx DSP to investigate the molecular signature of unstable plaques in coronary artery disease using FFPE samples collected post-mortem [52]. Combining histology and ST, the study showed that unstable plaques were enriched in proinflammatory and prothrombotic pathways such as interferon-gamma (IFN-γ), tumor necrosis factor-alpha (TNF-α), cytokine signaling, vascular wall interaction, and hemostasis. Interestingly, the intima and media also have differential gene expression in prothrombotic, proinflammatory, and cell stress pathways. In addition, CD68^+^ macrophage-like cells within unstable plaques displayed significant heterogeneity, including a subset of hybrid cells that have both smooth muscle and endothelial characteristics. 

The authors also investigated the transcriptional profiles of calcified and non-calcified unstable plaques. Smooth muscle cells from heavily calcified plaques were enriched in pathways related to amino acid metabolism and vascular signaling, while CD68^+^ cells from non-calcified plaques displayed upregulation of pathways related to stress response and extracellular matrix (ECM) regulation. Together, these findings provide insights into the regional transcriptional alterations present in unstable plaques, paving the way for preventive and therapeutic interventions. 

## 5. Current Limitations

Spatial transcriptomics has revolutionized the field, offering invaluable insights into human biology and disease mechanisms. However, several limitations exist, particularly when it comes to human samples. These limitations include cost, technical requirements, accessibility, sample availability, and data analysis.

### 5.1. Cost, Technical Requirements, and Accessibility

Spatial transcriptomics is a relatively new method that is resource-intensive. Generating data from tissue samples requires specialized equipment, highly trained personnel, and significant financial cost. These barriers limit access to ST, especially in places without the required infrastructure and personnel expertise. The cost of ST poses a significant barrier, further compounded by the rarity of high-quality human tissue, which leaves very small margin for error. In addition, there are limitations in terms of technical requirements that need to be addressed. The trade-off between resolution and sensitivity often constrains the ability to capture highly resolved spatial information while maintaining robust transcript detection. However, this is being rapidly overcome with new platforms that yield higher resolution and higher throughput. Furthermore, imaging-based platforms, despite being powerful, are time-consuming to process, thus limiting the number of samples that can be analyzed within a reasonable timeframe. The growing integration of multiomics approaches, such as pairing ST with scRNA-seq and proteomics, can overcome some of these challenges and provide more comprehensive insight into the complex biological mechanisms underlying cardiac diseases. 

### 5.2. Human Sample Limitations

The availability of high-quality human tissues remains a challenge. The main sources of these samples have been from patients undergoing open heart surgery, tissue biopsies, and explanted hearts. Large cardiovascular biobanks often rely on autopsy hearts, as freshly isolated hearts are rare. For instance, the Bruce McManus Cardiovascular Biobank in British Columbia is one of the few in Canada that collects such samples. Biopsies, on the other hand, present significant challenges due to their small sizes, and the priority is on patient diagnosis. Furthermore, RNA integrity preservation is a significant challenge for ST, especially in the context of explanted donor hearts, where variable ischemic times can subject RNA to degradation. This highlights the importance of tissue biobanks in standardizing and coordinating the sample collection and preservation process. Effective collaboration between heart surgeons, researchers, and biobanks is essential to streamline the workflow. Although ST was initially restricted to fresh frozen samples, recent compatibility with formalin-fixed, paraffin-embedded (FFPE) samples has broadened the range of usable specimens, making retrospective samples accessible. Currently, most ST platforms are primarily compatible with human and murine tissues, limiting their application to other organisms. Expanding compatibility to a broader range of tissues and organisms can open more doors for new areas of research. 

Another challenge with human samples is the significant heterogeneity among individuals. Existing work addresses this issue through several strategies. One common approach is the integration of scRNA-seq data with ST, where scRNA-seq serves as a high-resolution reference for consistent cell type and state annotation. This enables mapping and deconvoluting spatial spots to identify comparable cellular compositions among samples, despite individual variability. Researchers also use multiple biological replicates to assess reproducibility and similarities between datasets by calculating Pearson correlations of gene expression levels [43,45]. Furthermore, reference atlases, mostly from scRNA-seq data and, to a lesser extent, spatial data, provide baseline profiles against which individual sample variability can be measured. These approaches help address heterogeneity and allow reliable comparisons across samples. 

### 5.3. Data Analysis

The massive amount of data generated from ST and the complexity of data analysis is a major challenge for researchers without expertise in bioinformatics. Current analysis pipelines often require familiarity with programming languages (R/Python) and complex syntaxes [55]. Building on previous studies that have identified novel cell populations and niches, with data made available by the authors, there is an opportunity to develop computational tools that could automate the identification of these cardiac cell populations, similar to cellxgene [56]. Furthermore, these shared datasets could be used to train machine learning algorithms for pattern recognition, which might reduce the bioinformatic burden for researchers. Despite multiple analysis platforms existing, a streamlined and user-friendly one is currently lacking. Furthermore, with the large amount of data generated from each ST study, there is a need for a unified, open-source platform for data sharing and integration [57,58]. Such a platform would enable researchers studying similar topics to access and use shared data, thus fostering collaboration and accelerating discoveries. 

## 6. Conclusions

Spatial transcriptomics has become an essential tool for studying spatially resolved gene expression patterns, enabling major discoveries in understanding the complexity of human cardiac diseases. Our review highlighted the current technologies, particularly in commercially available ST platforms, to serve as a practical guide for researchers seeking to adopt ST into their studies. By employing ST technologies, multiple studies have uncovered spatial gene expression profiles that are critical for our understanding of human diseases, paving the way for the identification of novel therapeutic targets. Emerging platforms such as Stereo-seq, with its superior resolution and the ability to capture a significantly wider imaging area, or hybrid technologies such as GeoMX, which is selective in targeting specific imaging areas, further expand the range of ST applications in cardiovascular research. Ongoing innovations in ST technologies and platform design promise greater resolution, sensitivity, and efficiency, broadening the scope of ST applications. 

The integration of ST with multiomics approaches, such as scRNA-seq and proteomics, offers opportunities for a more comprehensive understanding of molecular mechanisms. Emerging spatially resolved proteomics, which enables researchers to visualize and quantify proteins while preserving the spatial context, holds significant promise for spatial-omics analyses. This comprehensive approach allows for the simultaneous mapping of gene expression and protein localization, providing a more holistic understanding of biological complexity. As these technologies continue to advance, they have the potential to transform our understanding of health and disease, paving the way for novel diagnostic and therapeutic strategies that address unmet clinical needs.

## Figures and Tables

**Table 1:** Key parameters of commercially available spatial transcriptomic platforms.

No	Platform	Method	Species Compatibility	Tissue Compatibility	Capture Area Size	RNA Targets	Resolution	Tissue Section
1	10x Genomics Visium	Sequencing-based	Human, mouse	Fresh frozen, fixed frozen, FFPE	6.5 mm × 6.5 mm	Whole transcriptome	Single cell, 2 × 2 µm	Standard glass slides
2	STomics Stereo-seq	Sequencing-based	Human, mouse, others	Fresh frozen	1 cm × 1 cm, up to 13 cm × 13 cm	Whole transcriptome	Subcellular	Stereo-seq chip slides
3	Curio Bioscience CurioSeeker	Sequencing-based	Human, mouse, others	Fresh frozen	3 mm × 3 mm or 10 mm × 10 mm	Whole transcriptome	Single cell	CurioSeeker tiles
4	10x Genomics Xenium	Imaging-based	Human, mouse	Fresh frozen, FFPE	22.5 mm × 10.5 mm	5000 genes	Subcellular	Xenium slides
5	NanoString CosMX	Imaging-based	Human, mouse	Fresh frozen, FFPE	20 mm × 15 mm	6000 genes	Subcellular	Standard glass slides
6	Resolve Biosciences Molecular Cartography	Imaging-based	Human, mouse, others	Fresh frozen, FFPE	8 placement areas, each area 1 cm^2^, in total maximum 26 mm^2^ across all samples	100 genes	Subcellular	Molecular Cartography slides
7	Vizgen MERSCOPE	Imaging-based	Human, mouse	Fresh frozen, fixed frozen, FFPE	3 cm^2^	1000 genes	Subcellular	MERSCOPE slides
8	NanoString GeoMx	Hybrid	Human, mouse	Fresh frozen, fixed frozen, FFPE	35.3 mm × 14.1 mm	Whole transcriptome	Area of illumination, minimum 5 μm × 5 μm	Standard glass slides

**Table 2:** Summary of studies using spatial transcriptomics in human cardiac tissues. FFPE, formalin-fixed paraffin-embedded; GeoMx DSP, GeoMx Digital Spatial Profiler; hPSC, human pluripotent stem cell; ISH, in situ hybridization; ISS, in situ sequencing; MERFISH, multiplexed error-robust fluorescence in situ hybridization; OCT, optimal cutting temperature; scATAC-seq, single-cell assay for transposase-accessible chromatin sequencing; scCITE-seq, single-cell cellular indexing of transcriptomes and epitopes sequencing; scRNA-seq, single-cell RNA sequencing; ST, spatial transcriptomics.

Topics	Author	Year	Tissue Type	ST Technology	Commercial ST Platform	Other Omics Technology (Platform)	In Vitro/In Vivo Verification
Cardiac development	Asp et al. [43]	2022	Fresh frozen	Sequencing-based	-	scRNA-seq (10x Genomics)	-
				Imaging-based (ISS)	-		
	Farah et al. [44]	2024	FFPE	Imaging-based (MERFISH)	-	scRNA-seq (10x Genomics)	Yes (hPSC model, mouse model)
	Lazar et al. [45]	2024	Fresh frozen	Sequencing-based	Visium	scRNA-seq (10x Genomics)	-
				Imaging-based (ISS)	-		
Cardiac electro-anatomy and immunology	Kanemaru et al. [46]	2023	Fresh frozen, OCT frozen, FFPE	Sequencing-based	Visium	scRNA-seq, scATAC-seq (10x Genomics)	Yes (hPSC model)
	Vyas et al. [47]	2024	FFPE	Hybrid	GeoMx DSP	scRNA-seq, scCITE-seq (10x Genomics)	Yes (hPSC model)
	Amrute et al. [48]	2024	Used previously published ST datasets			scRNA-seq, scCITE-seq, scATAC-seq (10x Genomics)	Yes (human fibroblast models, mouse models)
Ischemic heart disease	Kuppe et al. [49]	2022	Fresh frozen	Sequencing-based	Visium	scRNA-seq, scATAC-seq (10x Genomics)	Yes (immortalized human cell line, mouse model)
	Lina-Kuosmanen et al. [50]	2024	Fresh frozen	Sequencing-based	Visium	scRNA-seq (10x Genomics)	Yes (human vascular cell lines)
				Imaging-based (ISH)	Molecular Cartography		
	Ninh et al. [51]	2024	Used previously published ST datasets			-	Yes (hPSC model, mouse model)
	Gastanadui et al. [52]	2024	FFPE	Hybrid	GeoMx DSP	-	-
